# Organizing Social Citizenship for People Living With Dementia: A Community Case Study

**DOI:** 10.1177/14713012261471604

**Published:** 2026-07-30

**Authors:** Milan van Keulen, Josje Kok, Roland Bal, Anne Margriet Pot

**Affiliations:** 1 Erasmus School of Health Policy & Management, 6984 Erasmus University, Rotterdam, The Netherlands; 2 Health and Youth Care Inspectorate, Ministry of Health, Utrecht, The Netherlands; 3 Optentia, North West University, Vanderbijlpark, South Africa

**Keywords:** dementia, social citizenship, community, care-collectives, boundary work

## Abstract

This study explores a new clustered social housing concept for older people through the lens of social citizenship for people living with dementia. For people living with dementia citizenship occurs most evidently in the ordinary domestic and community spheres. Based on ethnographic fieldwork, including observations and interviews, our analysis reveals how the design of this concept supports creating care-collectives outside of the often prevalent medical view. A structural interprofessional meeting acts as an institutionalized coordination body which pro-actively monitors, coordinates and shapes collectives for residents to receive care and support and remain engaged with the community. Initiating care-collectives from signals (early changes in residents’ functioning and wellbeing) has potential for enacting citizenship for people living with dementia, by situating them in a broader effort to engage with, and be part of a community. However, in the cross-domain collaboration new dilemmas and ethical considerations arise around boundaries of professionals responsibilities, information sharing, and their amount of agency to tailor services for residents.

## Introduction

Despite growing attention to social citizenship in dementia, support practices remain structured around diagnosis and medicalized care (Campbell et al., 2016; O’Connor et al., 2022; Ursin & Lotherington, 2018; Yu et al., 2025), which in turn constrains opportunities for participation and the enactment of citizenship for people living with dementia in everyday community settings. At the same time, emerging housing concepts (e.g. clustered social housing) create new spaces where care, welfare, and community intersect (Hamers et al., 2024; Johnson, 2025; Smith et al., 2022), but there is still limited understanding of how citizenship can be enacted in such cross-domain settings.

Social citizenship for people living with dementia provides a normative and analytical lens to understand participation, recognition, and belonging beyond care needs: “It involves justice, recognition of social positions and the upholding of personhood, rights, and a fluid degree of responsibility for shaping events at a personal and societal level” (Bartlett & O’Connor, 2010, p. 37). The core idea of the ‘social’ in social citizenship is that citizenship for people living with dementia is not a passive status, but is shaped by active involvement and support from others to enact and realize citizenship (Bartlett & O’Connor, 2010; O’Connor et al., 2022; Ursin & Lotherington, 2018).

In order to realize social citizenship in practice, Ursin and Lotherington (2018) emphasize that citizenship for people living with dementia is collectively enabled by networks of relationships among informal caregivers and professionals from different domains, alongside socio-material agents. They call these networks ‘care-collectives’, and emphasize that work is required to create these social formations, as well as collaboration among various actors to enact citizenship (Ursin & Lotherington, 2018).

They describe four different mechanisms in care-collectives that support the enactment of citizenship for people living with dementia; invisible work (recognizing the often overlooked effort of unpaid workers in organizing everyday life), coordination work (the continuous attunement of different, including new, services, professionals and practices to stabilize the care-collective), flexibility (adaptability of services), and shifting between knowledge regimes (the medical knowledge regime is often dominant, but others like embodied knowledge are as important) (Ursin & Lotherington, 2018, p. 68).

Enacting social citizenship thus requires coordinated and flexible collaborative efforts across domains. However, this aspiration constitutes a complex undertaking in its own right, reflected in the vast literature on integrated care (Abrams et al., 2019; Exley et al., 2024; Lau et al., 2018; Österholm et al., 2022). Integration requires boundary work, referring to the work professionals do to set, overcome or navigate boundaries of professional responsibility, different organizational logics and administrative systems, in order to be able to work across domains (Tazzyman et al., 2021). As the lens of social citizenship reshapes the roles and responsibilities of different professionals involved in relation to people with dementia and family caregivers, boundary work is necessary to coordinate this change in practice, and mitigate potential frictions. However, little research has focused on this aspect (Baldwin & Greason, 2016; van Muijden et al., 2025).

Research on the practices of cross-domain collaboration shows that its effectiveness depends on the amount of agency professionals have to adjust or overcome these boundaries (Quick & Feldman, 2014; van Muijden et al., 2025). Horizontal boundaries, referring to the professionals’ agency to tailor services to the specific needs from a client’s perspective, determine the scope of possibilities; for example, a home care worker staying longer than his scheduled time to provide an extra task in response to a household support worker being absent (Abrams et al., 2019). The ‘discretionary space’ professionals have to tailor services is, in turn, greatly determined by vertical boundaries, for example by the financial structures that pay for services (van Muijden et al., 2025).

Next to the boundary work necessary to operate across domains in order to enact citizenship for people living with dementia, citizenship is also constrained by the construction of care-collectives, as they are predominantly formed by healthcare services, foregrounding a medical view (Ursin & Lotherington, 2018, p. 69). An explanation for this dominant focus may be that care-collectives currently only emerge when a person is diagnosed with dementia; the starting point therefore being a medical condition. Yet, for people living with dementia, a diagnosis is not where the experience with the condition starts (Campbell et al., 2016). On average, the time from the first experienced symptoms to a dementia diagnosis is estimated to be 3.5 years, and the percentage of undiagnosed dementia in Europe is estimated to be over 42%, varying widely per country (Kusoro et al., 2025; Yu et al., 2025).

Enacting citizenship for people living with dementia requires recognizing that participation in community life cannot be inferred from a diagnosis alone, as people’s lives and social roles both precede and exceed the diagnosis. Before a diagnosis is set, consequences associated with living with dementia, such as social withdrawal and increasing dependency on others, may already be at play (Van Gennip et al., 2016; Wijngaarden et al., 2019), thus also needing different types of care or support.

People living with dementia are primarily engaged in social life within their neighborhoods, which can either facilitate or obstruct their engagement with the environments through which their identity and selfhood are shaped (O’Connor et al., 2022; Ward et al., 2018). The WHO Healthy Ageing framework extends this perspective by emphasizing that people’s ability to participate in everyday life is not solely determined by individual mental and physical capacities, but is also shaped by environmental factors, including social, material, and organizational aspects. In this sense, social citizenship is not only socially enacted but also environmentally and structurally enabled or constrained (Pot et al., 2023; WHO, 2020).

The literature on social citizenship stresses the importance of seeing people living with dementia as engaged members of their community rather than primarily subjects of care, by separating citizenship from the often immediate presence of care in relation to dementia (Bartlett & O’Connor, 2010, p. 4). It further aspires for people living with dementia to live free from stigma and to grow and participate to their fullest extent possible (Bartlett & O’Connor, 2010). Broadening care-collectives beyond the dominant medical view and towards social citizenship requires the emergence of collectives despite a diagnosis, alongside broader coordination aimed at engaging with the community, an area that is still under-researched.

Together, the literature on social citizenship, care-collectives, and boundary work allow us to examine how citizenship as an ideal, must be practically enacted, and negotiated within communities by focusing on cross-domain organizational practices. In this study we aim to provide insight into the work this entails by studying clustered social housing, a relatively new site which offers an empirical and organizational setting where living, care, and community are intentionally combined. The clustered living concept is an increasingly adopted policy response to population ageing in the Netherlands, and resembles international initiatives like co-housing or supportive housing (Hamers et al., 2024; Johnson, 2025; Smith et al., 2022).

In the Netherlands, following the 2015 austerity measures aimed at reducing care costs, older people with low care needs were no longer eligible for residential care homes. Consequently, much of the responsibility for care and support shifted to home care and other community services. Several issues have since emerged, such as care fragmentation characterized by many small home care organizations operating with limited interaction, and a shortage of suitable housing for an increasing aging population (Alders & Schut, 2019; Dungen & Koesveld, 2018). Additionally, the Netherlands as of 2025 has an estimated 310.000 people living with dementia, of which 75% lives at home. By 2050, the number of people living with dementia is projected to have almost doubled to 610.000 (Alzheimer Nederland, 2025).

The national government stimulates municipalities and regional organizations towards new clustered living arrangements for older people. These arrangements are designed to have multiple grouped homes or apartment buildings suitable for older people (e.g. single story, wheelchair accessible, dementia-friendly design), with a shared meeting place, with care and social welfare close by (Dutch Ministry of Housing and Spatial Planning, 2023).

In this case study we address a gap in understanding how social citizenship is enacted in everyday, cross-domain organizational settings that are not structured around a dementia diagnosis. To address this gap, this study examines the following research question: how can everyday organizational practices within a shared community enact citizenship for the residents, including those living with dementia, and what dilemmas arise in doing so?

## Method

### Research Background and Setting

This study researched a clustered social housing arrangement in one of the larger municipalities of the Netherlands, organized in apartment buildings. The clustered housing arrangement provides private social housing with on-site care services delivered by dedicated staff from one long-term care organization, one social welfare organization, and opportunities for community engagement through organized activities and resident-led volunteering. Our fieldwork took place in one apartment building comprising around 240 homes, of which at the time of our fieldwork around 110 residents received some kind of care or support.^1^

Specific for this living arrangement was the fact that the municipality set conditions aimed at fostering an active, dementia-friendly community. For example, the housing corporation was required to maintain a mix of so called ‘providers and receivers’ – residents who wish to fulfill an active role in the community and provide support, and people who would like to participate, but may require some support in doing so. Also, all activities had to be accessible for people living with dementia, include a minimum amount of mobility/sport activities, facilitate shared meals, encourage and support residents to organize activities themselves if they wish, and include a yearly meeting targeted to recognizing and interacting with people living with dementia.

Other requirements include an income cap, to facilitate housing for people with a lower socio-economic status, and priority for applicants aged 65 and older, and a minimum of 55 years. Lasty, the municipality stipulated the housing association, care and social welfare organization to have the structural signal-meetings to foster collaboration and support the social-participation of all residents.

The main focus of this study were these recurring signal-meetings. In this meeting dedicated staff from different domains meet structurally and discuss the lives of residents in the apartment building, early changes in residents’ wellbeing, including participation, are discussed to enable timely, person-centered support, with the explicit aim of supporting their social participation and wellbeing. Importantly, these meetings are organized around broadly defined ‘signals’ – such as changes in participation or wellbeing – rather than from a medical diagnosis or formal eligibility assessment for support. These consistent, recurring meetings of care, housing and social welfare actors, organized around signals constitute an empirically distinctive case.

### Data Collection

Using a focused ethnographic approach we collected data by following the dedicated team of community workers – including staff responsible for community activities and services, as well as volunteers, care professionals and the housing corporation as they organized, facilitated and reflected on the daily life of residents in one apartment building (Knoblauch, 2005; Wall, 2015). Focused ethnography as a research approach uses shorter timeframes than more traditional ethnographic approaches but is especially suitable for gaining a detailed understanding of specific phenomena in emerging contexts, like the clustered living initiative (Knoblauch, 2005; Wall, 2015).

Our fieldwork took place from November 2023 to May 2024. The data collection started with roundtable discussions between the care organization present in this building and the first, second and last author. The first author, who has experience in ethnography in care settings and qualitative research with people living with dementia, was then tasked with developing a detailed understanding of the inner workings and underlying mechanisms of the clustered living concept (Rhodes, 2015). With this aim, the first author started with observations of signal-meetings, and from there shadowed the different relevant practitioners attending the meetings (case manager dementia, day care worker, community nurse), and observing a training in person-centered care for home care practitioners.

During the fieldwork all authors regularly reflected on the data collected. Fieldwork was concluded when all relevant organizational and professional aspects of the apartment building were sufficiently covered, and new observations did no longer generate new information. Near the end of the fieldwork, we identified a lack of in-depth information on the municipal perspective. The first author therefore conducted an interview with the project manager from the municipality overseeing and initiating this housing project. The first author also conducted a final interview with the community nurse, who chaired the signal-meetings, to validate our interpretations of the ethnographic material and discuss our preliminary findings. In total, this resulted in 35 hours of fieldwork. Table 1 provides an overview of the fieldwork activities.

**Table 1.** Overview Fieldwork

Method	Source	Hours	Total
Shadowing	Case manager dementia	3 h	9 h
	Community nurse	6 h	
Non-participatory observation	Bi-weekly signal-meeting	6 h	17 h
	Training person-centered care for home care staff	2 h	
	Day care activities	9 h	
Interviews	Community nurse	1 h	2 h
	Project manager municipality	1 h	
Roundtable discussions	Professionals from participating care organization and authors		7 h
Total			35 h

### Data Analysis

During observations, fieldnotes were made and where possible, direct quotes were written along. Immediately afterwards fieldnotes were digitally worked out in more detailed ‘thick’ account of our case study, capturing not only observed behavior but also relevant contextual information (Emerson et al., 2011; Geertz, 1973). Interviews were audio-recorded and transcribed verbatim. All data were converted into textual form and analyzed using Atlas.ti software by the first author. Materials were read, discussed and reflected upon in meetings with all authors to develop a shared understanding of the practices under study and to inductively identify initial themes. In a second phase, the data and codes were revisited, resulting in descriptive categories that captured recurring accounts and organizational practices. In order to make sense of the categories we then turned to the literature to search for ways to interpret our findings. This abductive approach let us to use the concepts of care-collectives and boundary work, which helped us to better understand the practice of the signal-meetings (Tavory & Timmermans, 2014; van Hulst & Visser, 2025). The literature on care-collectives for example helped us in understanding the signal-meeting more conceptually as different domains being collectively responsible for forming the daily life of residents, outside the medical regime. Boundary work helped us in finding a vocabulary and understanding for the interprofessional work that was being done within the signal-meetings. With these concepts we started coding more deductively. In the last step we identified three main dilemmas in these categories underlying the daily practice. This study followed the relevant EQUATOR guidelines for qualitative research (SRQR) (O’Brien et al., 2014).

## Ethical Considerations

The ESHPM Research Ethics Review Committee granted ethical approval for this research under reference number ETH2122-0419. An information letter on the research was sent to both the professionals, and community residents encountered while shadowing, before entering the field. The research team received verbal consent from all participants.

## Findings

We start this section with a descriptive account of how the clustered living concept and signal-meetings function as an organizational practice, before turning to the dilemmas that arise in their everyday functioning.

### Assembling a Broader Collective for Preventive Action

The participants of the signal-meeting vary depending on availability, but the permanent team consists of representatives from three organizations. The care organization is represented by multiple professionals: the community nurse, who chairs the meetings, the case manager dementia, the daycare manager and the home support worker (who supports and coaches independent living and social participation). The housing officer represents the housing association, and the community worker represents social welfare.

The signal-meeting, we found, operates as a coordination body establishing relations to enable community engagement and support. Within the clustered apartment building, the different parties have direct lines of communication and are familiar of each other’s roles, expertise and organizational logics. Signals discussed operate as ‘calls to attention’ observed in professionals’ own practice or passed on from residents or other professionals not attending the meeting. Signals address situations which other professionals need to be updated on and if deemed necessary, act on. For example, when a community worker is made aware of a resident lashing out during an organized activity for residents, which influenced the safe atmosphere of the group, she informs the case manager dementia to check-in with this resident, to see if the current arrangements are still suitable. Professionals updating each other informs and facilitates them to work pro-actively, for example by creating new networks of relations within the community by connecting residents:

As the signal-meeting proceeds, attention returns to the home support worker, who raises another concern. She shares that a woman is becoming increasingly isolated, and she asks if there’s someone, a volunteer, who can take her downstairs [where the activities are organized]. The community worker shares that her neighbor is very sociable and a volunteer. She’s from the Creative Arts Club, and she has already connected the two residents. She says she will ask the neighbor to take the woman to the Creative Arts Club on Monday and downstairs to the weekly activity on Wednesday. [observation notes 30-11-2023]

The signal-meeting thus operates as a coordinating body, pro-actively facilitating relations between professionals, volunteers, and residents, and linking them to activities.

We found that the signal-meeting also nourishes and shapes the community. As illustrated in the observation note above, bringing residents together enables further social connections within the community. This work is further extended by publication of a monthly newsletter, creating a shared identity, and addressing residents as part of the community. Stipulated by the municipality, the newsletter informs the community on upcoming (resident-led) events and provides guidance for residents where they can seek additional information or support (e.g. computer support from a volunteer or dedicated consultations hours for care and social support related questions).

The social cohesion and interaction between residents are supported by organized information meetings, like a yearly meeting on how to interact with people living with dementia, and by being actively involved in renovations by means of an advisory group. The work of the different professionals from different domains thus entails more than discussing signals to increase needed support or community engagement. It also feeds the community with incentives to actively participate and connect, informing the community, and educating residents on dementia.

### Dilemmas in Working Across Medical, Social Welfare and Housing Domains

The analysis of this organizational practice revealed three dilemmas that, illuminate tensions, barriers and ethical questions, in relation to the pursuit of social citizenship.

#### Dilemma 1: Exchanging Information, a Balancing Act

As illustrated above, the signal-meetings are used as a place for information transfer and coordinating networks of relations that contribute to the life of residents in the community. However, being from different organizations forces professionals to perform boundary work to circumvent privacy laws constraining what they (feel) they can disclose. Some information about residents is shared within professional confidentiality, involves medical data, or it is uncertain what information can be shared between organizations. An example of the boundary work is that privacy laws from each organization makes that the names of residents cannot be used. In wanting to circumvent this, the professionals refer to residents not by name but house number.

The amount and type of information attendees at the signal-meetings have about a resident is also distributed. Professionals from the care organizations have more medical background information on the resident than, for example, the housing association representative, who in turns knows more about nuisances amongst residents. Inadvertently, professionals who know more about a particular resident moderate the narratives of residents as well as the decision-making process, by deciding what to share:

The community nurse explains to me [first author] that she often finds it difficult to determine what she can and can't share based on the VGA [General Data Protection Regulation]. She often knows more than is being discussed, but she doesn't consider it necessary to share someone's entire life story. (…) “The question then becomes how much do I have to share to achieve the goal” she says. At the outset of the signal-meetings, she tended to share a bit more information in order to build trust amongst the parties**.** [observation notes 21-03-2024]

In the signal-meeting, privacy was often an invisible attendee – raising questions, creating practical barriers, and prompting ethical considerations. Professionals ponder if the information they have needs to be shared for enabling better decision-making, can be shared, or if someone’s privacy and dignity should be safeguarded. Concurrently, not being able to share medical information may also have a destigmatizing effect by shying away from medical interpretations. On the other hand, medical information can also determine what kind of support or arrangements are appropriate or not. Underlying this practice thus lies a broader question ‘what information needs to be known collectively, to make the right decision?’ Each attendee interprets this question individually when deciding what to share, balancing collective insights with ethical considerations. Moreover, sharing information whether it is allowed or not, also functioned as a way to build trust between parties and this then helps in attending to signals in more productive ways.

#### Dilemma 2: Ambiguous Responsibilities

Another dilemma we found in our observations and interviews concerns how far the responsibility of the professionals reaches or should reach. An often-recurring example illustrating this issue was loneliness amongst residents. When loneliness was discussed in the signal-meetings it was unclear who exactly should be addressing this, from which professional mandate, and how far their responsibility in trying to overcome the loneliness experienced by residents reaches. The community nurse explained:

“(…) I have a woman here [in the apartment building] who doesn't leave her house, and that's causing a lot of concern. Well, who’s problem is that? I've really tried my best to understand her needs. But some needs I can’t fulfill, but then we do have to accept that she’s just sitting there. (…) We [as professionals] are so wired to want to prevent everything, and sometimes that's simply not possible. (…) You constantly have to stand beside them [residents], trying to see through their eyes. And if we start to assume someone is lonely or needs to leave their home then we're actually preoccupied with what we think, or whether it meets our social norm. And then you're really no longer connected to that person.” [interview community nurse]

Intervening with a resident’s life is not always a neutral response. It can also impose a normative professional ideal. Professionals in this case must reflect whether the situation at hand is part of their responsibility (and where this responsibility ends), or is an autonomous decision by a resident to shape their own life. As the community nurse explains, problematizing a situation from a professional’s social norm, can also undermine citizenship by imposing a norm conflicting with that of the residents. Furthermore, professionals active in the community have to negotiate issues which are not institutionally embedded amongst the different domains and reflect per case whether to meddle with or accept a situation. Because there are no definite answers, different professional opinions conflict when discussing such cases:

The home support worker brings in another signal: a woman might need extra help, a staff member who occasionally visits her thought so. She’s very lonely. The housing officer responds: “But we don’t do that, loneliness. What exactly does she want? She goes to IKEA alone every week, and she recently visited her niece.”

(…) The home support worker says: “I think this is a bit of an issue. She requires complex support, and we don’t have volunteers for that, but a person needs to have 5 contacts a week.” “Well, that varies from person to person.” the community nurse in training says. “My mother has 30 contacts a week and still feels lonely.” The housing officer responds in disagreement. The home support worker responds: “but sometimes we don't have volunteers, and then we just conclude that loneliness is not our job?” [observation notes 08-02-2024]

Cases that lack institutional embeddedness, or lie outside of available resources – such as reliance on community volunteers – often meander on without a clear solution. In order to enact citizenship in these situations, professionals must operate within these ambiguous boundaries of responsibilities and norms and discuss their responsibility towards the issues at hand, without clear answers. As the observation note shows, this can create friction when professionals disagree. More importantly for social citizenship is that this discussion also requires involving the person in question, to determine what they prefer or prioritize, instead of deciding for them. This brings us to the third dilemma.

#### Dilemma 3: Broadening the Scope of Care-Collectives

The last dilemma we found considers organizing the scope of the coordinating body. Signals are largely shared from the different professionals’ practices. Which and what kind of signals are shared is thus determined by which professionals are coordinating. This raises the question of which perspectives should be included, and in what way. We distinguish this dilemma into two dimensions: the inclusion of other professionals and the inclusion of residents.

The assemblage of different parties in the signal-meeting forms the framework in which signals will be understood. The scope of the signal-meeting is currently limited to care, housing and social welfare, but other organizations operating in the community like local transport services, local stores and mental healthcare are missing perspectives:

The housing officer shares with the other professionals that what she finds the most difficult about the integral collaboration, is that some residents receive support from mental healthcare organizations, which she does not know about. Although she might want them to join, it would be very difficult as this support is provided by many different organizations. [observation notes 11-01-2024]

Professionals in the signal-meeting are often not aware if residents receive support from or are dealing with other parties such as mental healthcare. The current participants of the signal-meeting are obliged to join by the municipality, as stipulated in its tender requirements of the clustered living initiative. Inviting other parties often meets resistance regarding tight schedules: “Other parties find it difficult to justify the time investment. They don’t understand that it doesn’t cost 1 hour, but yields two” [Housing officer, observation notes 14-12-2023]. Professionals face a vertical boundary in not having the mandate to stipulate other organizations to join, while missing their perspective in coordinating signals.

#### Including Residents

Increasing the diversity of professionals present at the signal-meeting can mitigate horizontal boundaries and increase coordination work, but decisions are still made by professionals about residents, without the residents themselves being involved. Their needs and wishes are conveyed through others in the meetings, an issue the professionals are aware of and struggle with:

“We first consciously decided not to do that [invite residents], but I think that’s precisely the need of the residents. When I speak to them, it’s very strong, the need to be included, to think along and participate. That’s where we make the plans, that’s where we map things out, so they just belong there. So yes, I miss that. Looking back now, I think, we didn't handle that well.” [interview community nurse].

As the signal-meeting – and the clustered living initiative as a whole – is a relatively newly formed initiative, professionals are still experimenting with and searching how to actualize its goals, and with whom. At the onset, the different parties were not aware of each other and had to get to know and trust each other, therefore not inviting residents from the start. Now realizing that it is of utmost importance for residents, we witnessed that the professionals are starting to invite them. Being able to continuously reflect and address recurring issues and adjust the design of the signal-meeting accordingly is crucial to further the development and use of the meetings for enacting social citizenship.

## Discussion

The enactment of social citizenship is a collective responsibility, often supported by care-collectives - networks of relations among (socio-material) agents - but it remains shaped by medicalized knowledge regimes that may obstruct the focus on social citizenship (Bartlett & O’Connor, 2010; Ursin & Lotherington, 2018). Less is known about how such processes can unfold in everyday, cross-domain settings that are not structured around a dementia diagnosis.

Our findings show that a recurring, stipulated and cross-domain collaborative meeting proved helpful for professionals to coordinate and form care-collectives for residents, allowing the formation of different types of collectives, outside the dominant medical regime. By proactively discussing residents’ functioning and wellbeing from different professional realities (social welfare, housing, home care), early signs of a decrease, like social isolation or increasing dependency of residents, can be identified and addressed independently of a formal diagnosis of dementia (Campbell et al., 2016; WHO, 2020).

We conceptualize the signal-meeting as an institutionalized form of coordination work, in which the different parties involved continuously establish, coordinate and evaluate care-collectives. This way of organizing care-collectives within a community facilitates enacting citizenship for people living with dementia, by situating them in a broader effort to engage with, and be part of a community (Beard et al., 2016; O’Connor et al., 2022; WHO, 2020). Citizenship is thus actively organized through the ongoing interprofessional coordination of socio-material agents in the community (Ursin & Lotherington, 2018). A notable difference between care-collectives as originally described by Ursin and Lotherington (2018), is that the emergence of networks of relations is proactively realized through coordinating work between domains, based on signals of decline in functioning or wellbeing of residents, instead of emerging from a diagnosis (Ursin & Lotherington, 2018).

Other mechanisms Ursin and Lotherington (2018) describe for care-collectives that enact citizenship are flexibility, switching between knowledge regimes and acknowledging invisible work. These mechanisms were also evident in our analysis of signal-meetings. Having a signal as starting point facilitates the navigation between different knowledge regimes and domains of action, and beyond the dominant medical regime. Also, the starting point for a discussion being a signal foregrounds invisible work needed or done by others, as the threshold for a collaborative discussion is low. This further increases professionals’ awareness of each other’s work and facilitates flexible arrangements as small non-formalized arrangements are easily made, for example by asking a resident volunteer to guide another resident to community activities.

The proactive engagement of dedicated staff of cross-domain parties, situated in one community, provides a fruitful organizational practice for enacting social citizenship for residents in a community. As social citizenship requires active involvement of others, the stipulated signal-meeting provides a collaborative space where citizenship for the residents in the demarcated community (a single apartment building) is organized. It provides insights in how to sustain a sense of community by facilitating social networks and support systems for (and by) residents and keep residents engaged without needing a medical diagnosis as starting point (Odzakovic et al., 2021; O’Connor et al., 2022; Ursin & Lotherington, 2018; Ward et al., 2022; WHO, 2020). However, it also raises new practical and ethical dilemmas. We identified three such dilemmas.

The first dilemma concerns the practical hurdles of communication in cross-domain collaboration on an operational level. Professionals from different domains and organizations must continuously weigh what they ought to share to facilitate coordination work, and what they cannot share as it might violate privacy requirements. Professionals from different domains are compelled to perform horizontal boundary work in trying to avoid potential violations in their inter-organizational communication (Lau et al., 2018; Tazzyman et al., 2021; van Muijden et al., 2025). But, more importantly, it also raises an ethical and epistemic question regarding how much and what kind of personal information needs to be shared, and with whom, in order to organize citizenship. Social citizenship broadens the dementia framework as more than a medical condition and stimulates the focus on identity (Bartlett & O’Connor, 2010; O’Connor et al., 2022). However, it remains unclear for professionals (amidst reluctance to share information due to uncertainties surrounding privacy legislation) what specific knowledge about residents supports the organization of citizenship.

The second dilemma we found is related to the ambiguous boundaries of responsibility in organizing citizenship. In enacting social citizenship, topics of discussion are focused on stimulating, for example, residents’ engagement with the community or loneliness, issues that lack institutional embeddedness (Baldwin & Greason, 2016; O’Connor et al., 2022; Tazzyman et al., 2021). Enacting social citizenship for people living with dementia thus operates within an area of ambiguous responsibilities, social norms, and professional boundaries, which professionals must navigate amongst themselves. While re-envisioning professional roles towards social citizenship might help in bringing more clarity in responsibilities (Baldwin & Greason, 2016), we found that the division of responsibilities is also related to the framing or interpretation of a specific signal of a resident. As such, negotiations within care-collectives will always be necessary in practice.

This dilemma does not only address professional responsibility of who should be doing what, but also the boundaries between imposing a normative ideal of an interpretation of the goal of care on the one hand, and autonomous decision making about one’s own life on the other. Professionals continuously consider the boundary of where their professional responsibility is exhausted in discussing issues regarding social citizenship, like community engagement. Although the topics requiring decisions may be new for professionals in the signal-meeting, the autonomy of people living with dementia should still be central in finding this boundary (O’Connor et al., 2022; Stumpf et al., 2025). The shift of seeing people living with dementia not as passive care recipients but as citizens, stresses this point already, but policy narratives and training could further support this change (Bartlett & O’Connor, 2010; Nedlund & Nordh, 2015). Addressing and discussing people’s wellbeing as residents, without being able to share medical background information across domains, may inadvertently facilitate a stigma free discussion (Bartlett & O’Connor, 2010). However, it still does not include people living with dementia in decision-making themselves, part of the third dilemma.

The third dilemma addresses the question which knowledge regimes should be represented in a coordinating body that form these collectives. A crucially important knowledge base currently missing, is that of the residents themselves. Although they are informed about and sometimes activated as a result of signal meetings, their perspective on their signals is important in order to mitigate speculation on needs and sustain autonomous decision making (O’Connor et al., 2022; Stumpf et al., 2025). The signals discussed are largely determined and made sense of by the stakeholders present, risking epistemic injustice when the voices of people living with dementia are not included (Fricker, 2007).

Broadening the scope for coordinating and creating care-collectives means that a large number of professionals can be involved, but also facing new practical challenges. To circumvent that certain perspectives or signals are not missed, the professionals of social welfare, housing and care, try to connect with other parties operating in the community but often face difficulties in including other organizations. The municipal conditions stipulating collaboration thus function as an important prerequisite for this coordinating body, by shaping the frame through which signals are interpreted (Nedlund & Nordh, 2015). In addition, users of the public services discussed can act as a catalyst in collaboration amongst parties, contingent upon the extent to which they are empowered and able to contribute their unique knowledge (Callens, 2023).

The three dilemmas presented further complicate the four mechanisms of Ursin and Lotherington (2018), and provide insights into the cross-domain organizational practice needed to enact citizenship for people living with dementia. Coordination work, we found, also entails negotiating responsibilities between professionals and considering whether their efforts are in response to a residents need, or an abstract ideal. This consideration also relates to invisible work. Because coordination is initiated around a signal instead of a diagnosis, invisible work is foregrounded, but it also elicits professional responsibility for issues of which it is unclear where the responsibility lies. While the coordination body enhances flexibility by enabling the different parties to easily adjust and attune their services, the convergence of organizations and knowledge regimes also creates obstacles. Professionals constantly walk the line regarding what information they can and should share collectively, in pursuit of the best outcomes for residents. As such, the signal-meeting should be seen as an evolving platform, calling for periodic reflections on the faced dilemmas and boundaries. Working on social citizenship means crafting the collectives that can sustain these as well as the conditions for those collectives to manage the dilemmas they are faced with.

## Strengths & Limitations

The goal of this study was to analyze the workings of a new residential concept as a lens for rethinking how communities can be designed to be dementia-friendly while actively supporting citizenship for all residents, including those living with dementia. In our analysis, we did not have the opportunity to include the perspectives of people living with dementia. While we describe this as an organizational reality professionals have to cope with, it is important that future research on this or similar concepts, focuses on the phenomenological perspective of residents (living with dementia) to understand their views and experiences towards this concept. A strength of this study is that it provides insight into the practical work, discussions and dilemmas involved in everyday realities of professionals across domains as they seek to support residents in the communities in which they work. The Dutch clustered living concept provides practical policy suggestions that can potentially further the enactment of social citizenship for people living with dementia, with stipulated cross-domain collaborations and signals as entry point for coordinating care-collectives.

## Conclusion

This ethnographic community case-study has provided insight into a new way of organizing the active involvement of professionals in enacting social citizenship for people living with dementia, as part of a community. A recurring interprofessional meeting between housing, care and social welfare acted as a coordinating body, in creating new care-collectives for support based on early changes in residents’ functioning and wellbeing. The interprofessional nature of the signal meeting, combined with initiating discussions based on shared signals of change, facilitates the formation of care-collectives beyond the dominant medical knowledge regime. At the same time, it raises new dilemmas regarding professional and institutional boundaries, requiring ongoing reflection and attunement.

## Data Availability

The research data generated during this study are, due to privacy and ethical consideration, not publicly available.*

## Note

1. Considering privacy and traceability we do not provide the exact numbers.
